# Severity and Temporality in Healthcare Priority Setting – A Case for A Condition-specific Affectable Time-neutral Approach

**DOI:** 10.1007/s10728-024-00493-z

**Published:** 2024-10-24

**Authors:** Lars Sandman, Niklas Juth

**Affiliations:** 1 National Centre for Priorities in Health, Department of health, medicine and caring sciences, Linköping university, Linköping, Sweden; 2 Centre for Research and Bioethics, Department of Public Health and Caring Sciences, Uppsala university, Uppsala, Sweden

**Keywords:** Priority Setting, Healthcare, Rationing, Severity, Fair Allocation

## Abstract

Priority setting of scarce resources in healthcare is high on the agenda of most healthcare systems implying a need to develop robust foundations for making fair allocation decisions. One central factor for such decisions in needs-based systems, following both empirical studies and theoretical analyses, is severity. However, it has been noted that severity is an under-theorized concept. One such aspect is how severity should relate to temporality. There is a rich discussion on temporality and distributive justice, however, this discussion needs to be adapted to the practical and ethical requirements of healthcare priority setting principles at mid-level. In this article, we analyze how temporal aspects should be taken into account when assessing severity as a modifier for cost-effectiveness. We argue that when assessing the severity of a condition, we have reason to look at complete conditions from a time-neutral perspective, meaning that we take the full affectable stretch of the condition into account without modifying severity as patients move through the temporal stretch and without discounting the future. We do not find support for taking the ‘shape’ of a condition into account per se, e.g. whether the severity has a declining or inclining curve, or that severity is intermittent rather than continuous. In order to take severity seriously, we argue that we have reason to apply a quantified approach where every difference in severity should impact on priority setting. In conclusion, we find that this approach is practically useful in actual priority setting.

In resource constrained healthcare systems trying to make ends meet, priority setting is constantly on the agenda. As priority decisions become more challenging, a solid ethical basis for the justification of such decisions is crucial. One factor playing a major role for priority setting in many healthcare jurisdictions is the severity of the condition. Countries like Sweden, Norway, the Netherlands and recently also England through NICE are explicitly referring to severity as a central factor to balance cost-effectiveness in their legal or semi-legal frameworks for priority setting in healthcare [1–4]. At the same time, despite the central role of severity, it has been noted that the concept lacks a normatively robust conceptualization, and several aspects of severity are still contested [5]. One such aspect is how severity should relate to the temporality of an ill-health-condition. How severe an illness is might be affected by how bad off that person is over time, but also their life-expectancy, i.e., how long the person lives^1^. In the philosophical literature on distributive justice, the issue of temporality has been on the agenda for a long time [6–10] and the main controversy seems to have been between positions arguing that we should only have a complete lives perspective (see for example [6–8]), or that such a perspective should be complemented by some more time-relative perspective, e.g. a forward-looking perspective or other time-relative perspectives [6, 8, 11, 12]. This discussion has been analyzed from the perspective of individuals being the relevant claimant when it comes to distributive justice, in some discussions explicitly stated (see for example Temkin [7], Ottersen [8] and Herlitz [12]) and otherwise implicit, by using individuals in the arguments. However, consider the following case in which severity would be used a modifier of acceptable cost-effectiveness:

Example 1: There is a new drug D_X_ with market approval for the rare disease X at T_0_ and it should now be decided whether D_X_ should be prioritized for patients with X. X is a genetic disease, with symptoms of degenerating muscles starting to show from age 10 and onwards in different degrees. Since X also affects lung muscles, the patients are destined to die at a fairly early age ranging from 25 to 45. Furthermore, the disease might also affect the patients’ immune systems, making them prone to also experience a number of co-morbidities of different sorts. At T_0_ the patient population (*n* = 100) will consist of patients in different ages, where symptoms started at different ages, and with different co-morbidities. On top of this, they will also have a number of health issues, unrelated to X, during their lives. If severity is a modifier for how much we are willing to pay for D_X_, what is the severity for this patient population?

If we assume individuals to be the relevant entity whose severity we should assess, the severity in this group will be highly heterogenous, regardless of which perspective we take. With a complete life perspective individual patients will be affected differently, since they live differently long lives with different co-morbidities impacting on their health-related quality of life (HRQoL). With a forward-looking perspective from T_0_ and onwards, individual patients will fare differently in terms of impact on HRQoL and life-length. With different other time-relative considerations, patients will be in high number of different situations when the decisions is to be made. Hence, it seems impossible to pinpoint severity in a way that will work for decision-making in this situation, regardless of which perspective we take. An alternative would be to focus on the condition X, and looking at real-life priority decisions, this is how it is done. That is, a priority decision is made on whether we should introduce D_X_ for the condition X. Hence, we need to know the severity of the condition rather than for each individual suffering from X.

Our aim in this article is to normatively analyze different ideas on temporality and distributive justice and how they can be applied to the severity, as a modifier of acceptable cost-effectiveness levels for which treatment to introduce to treat ill health *conditions*. This in order to contribute to a more robust conceptualization of severity as a central factor in practical priority setting within healthcare.

## Some Background Assumptions

In this article we will start from a number of background assumptions and limitations. The severity of a health condition might have different meanings or uses dependent on context. We might talk about severity in a more general sense, without referring to it as a basis or grounds for *distributing scarce resources* in health care (see for example [13]). We do not take a stand if such a more general concept can be distinguished from our focus of interest but allow for this possibility. Our focus of interest is however on the use of severity in healthcare priority setting. In this context, generally, severity is a pro tanto reason to prioritize patients or patient groups in health care. What exactly this implies in terms of resource distribution might be open to interpretation, dependent on what we interpret a higher priority to imply (see [14]). A more specific use of severity in priority setting is as a modifier for how cost-effective an intervention needs to be in order to be reimbursed [15]. Once again, whether this specific use of severity is equivalent to severity in a broader sense, we will leave open in this article. Our business is to look at this more specific use of severity, since this will have a crucial impact on the introduction of new pharmaceuticals (and other new technologies) within healthcare systems. To exemplify, in the Swedish healthcare system, if a drug targets a common condition with very high severity, the accepted cost-effectiveness threshold amounts to 1 000 000 SEK/QALY (equivalent of 80 000€/QALY), with lower thresholds for severe and moderately severe conditions [16]. Hence, the severity assessment might make the difference between reimbursement and not.

Severity as an equity modifier of acceptable cost-effectiveness thresholds has been on the agenda for a long time (see for example [15]). In distinction to the general dictum within health economics, that ‘a QALY^2^ is a QALY is a QALY’ – introducing severity into the equation implies that a QALY is more morally valuable in fulfilling equity requirements if provided to a more severely ill patient or patient group than if it is provided to a less severely ill patient or patient group (ceteris paribus). However, when explicitly looking at the jurisdictions using severity as such a modifier, we find differences when it comes to how severity relates to temporality. The Norwegian parliament decided in 2015 to interpret severity in terms of absolute shortfall, implying that severity equals the loss of QALYs for a patient population in relation to the normal population of the same mean age as when treatment should be administered [2]. This implies a forward-looking perspective on severity from the time of treatment in a condition’s trajectory (even if the original suggestion was a complete lives perspective, something which needed to be changed due to public discussion) [17]. In The Netherlands, as so-called proportional shortfall-approach is used, implying that severity is interpreted in terms of the share of the absolute shortfall in relation to the future possible QALYs without the condition or disease [3]. Once again, this voices a forward-looking perspective, but where the future loss is not simply aggregated (as with absolute shortfall), thereby making it less age-dependent. For England (and Wales) it is suggested that NICE use both absolute and proportional shortfall but also applying a discount rate to this assessment, voicing a specific take from a temporal perspective [4] Sweden, finally, have a more varied picture, where the temporal stretch of HRQoL impact has been assessed, but where premature death has been conceptualized in terms of time to death rather than impact on life-expectancy [18]. This, in order to avoid taking chronological age into account given legally established priority principles. A recent suggestion from the Swedish Dental and Pharmaceutical Benefits Agency, however, advocates that this should be complemented with an absolute shortfall assessment [19]. In effect, this shows a lack of uniform approach when it comes to severity and temporality and hence the need for further analysis^3^.

One contentious issue concerning severity is which currency to use (5). Since we want to avoid this issue here, we will simply assume that severity is related to both an impact on health-related quality of life (HRQoL) and life-length of a patient or patient population. In line with this, we will not take a definitive stand on what the exact currency of HRQoL is, e.g., what exact mix of subjective experiences, functional abilities, or other factors to take into account. In line with the QALY-measurement, to illustrate HRQoL in our examples, we will use a scale, where 0 indicates the lowest possible HRQoL, and 1 is our reference level for HRQoL where a person cannot be said to have any quality-related severity at all. Life-length will be illustrated in years, and we might then calculate an aggregated severity in terms of loss of (HRQoL X life-length) or for simplicity, QALYs, in relation to the reference level.

Finally, in line with the current practices in countries where severity is used as a modifier of cost-effectiveness (e.g. Norway, Sweden, The Netherlands etc.), we assume that severity is not a relative concept where the severity of a condition is determined in relation to where other people or patients are situated in terms of health-status. Rather, we assume that the rationale for taking severity into account is to give priority to worse-off-ness in relation to some absolute reference level where there is no severity and thus no reason to provide priority to people based on worse-off-ness.

In the article we will start with a brief summary of the discussion on distributive justice and temporality to provide a starting ground for our discussion. We then explore whether we should have a complete condition approach or a forward-looking approach on conditions, first only focusing on HRQoL impact and then also taking life-length impact into account. We conclude that we have reason to adopt a complete conditions perspective but only taking into account affectable stretches of time. In the rest of the article, we explore different suggestions for shape of conditions making a difference to the assessment of severity, ending up in the conclusion that shape does not matter. The article ends with conclusions and some reflections on its use in practice.

## Ideas on Distributive Justice and Temporality

As mentioned in the introduction there is a rich literature on distributive justice and temporality. To provide a starting point for our analysis we will focus on three different perspectives on this, drawing on two recent articles by Davies and Herlitz [11, 12], since they provide a good overview of these alternatives.

Traditionally, many philosophers seem to have arrived at the conclusion that we should have a complete lives view when assessing distributive patterns and whether they are considered fair or just (see for example [7, 8]). A classic argument against this view can be illustrated by the following example (using our HRQoL scale) (see for example [6]). Let us consider two individuals, A having half his life with a HRQoL of 1 and then half his life at 0,5, B has a level of 0,5 for half his life and then the rest of his life at 1. A and B lives equally long lives. According to the complete lives view, A and B lives equally good (or bad) lives, and the inequality between A and B the first half of life is compensated by the fact that they switch places for the rest of their lives. Now, it seems strange to claim that B can be compensated for the first inequality, by switching places with A the latter half of life. Instead, we have two different stretches of life that are unequal to the same degree. Those who are convinced by this argument have generally argued that the complete life’s view needs to be complemented with other, more time-relative, views (to use Davies’ term [11]).

Davies explores time-relative perspectives on justice and temporality, defining time-relative views like this:

A time-relative view says […]; our claims to benefits and against losses vary according to our situation when those benefits and losses will be felt [11]. , p. 465.

Furthermore, he claims that such views can complement a complete life’s view in different ways:

A time-relative principle can interact in various ways with a lifetime principle: it can act as a *constraint*; it can operate on an *equal* basis in terms of weighting people’s claims; it can itself be *constrained* by lifetime principles; it can act as a trump in a lifetime distribution; or it can act in different ways depending on the *context* [11]. , p. 465.

However, Davies does not take a definite stand on which pluralistic theory we should adopt.

Herlitz proposes a specific combination of the complete-life’s view with a forward-looking view, where priority is given to the future impact on life, even if the complete life will also have a certain weight [12]. Herlitz does not go into exact detail about how these two perspectives should be balanced. Rather, he points to a number of unresolved questions. Herlitz also see the need for arriving at a more practically useful conceptualization of severity, which we will try to make our business in the rest of the article.

Based on this discussion we identify three different principled approaches to explore in the following, modified to look at conditions, rather than individuals:

- We should consider the full impact of the condition over time on the patient population, regardless of when the treatment is considered (what we can call the complete conditions view).
- We should consider the future impact of the condition on the patient population, from the phase in the trajectory of the condition when the treatment is considered (what we can call the forward-looking conditions view).
- We should consider time-relative impacts of conditions on the patient populations, even if not affecting the overall impact from a complete or forward-looking perspective (what we can call the time-relative conditions view).

Or some combination of these. Let us explore these in the following.

## The Complete Conditions View vs. The Forward-looking Conditions View

### Temporal Impact on HRQoL

Let us start by focusing on conditions that imply a lowering of HRQoL but have no impact on life-length. On the complete conditions view, we should consider the full impact of a condition on a population’s life, regardless of when in time we are considering administering treatment to affect this condition. Hence, take a look at example 2:

Example 2: There is a new drug D_Y_ with market approval for the disease Y at T_0_ improving HRQoL to 0,7. Y is a disease, with reduced HRQoL from 1 to 0,5 at age 50 with a life-long impact, if nothing is done. The (average) life-expectancy of this population at age 50 is 35 years.

With a complete conditions view, the severity in this case will be 0,5 × 35 (impact on HRQoL X number of years in the condition) = 17,5. Given this, regardless of when D_Y_ will be administered to reduce the suffering of patients with Y, we should apply the same cost-effectiveness threshold.

Drawing on the classic counterargument against the complete life’s view, consider the following situation:

Example 3: There are two conditions A and B. A implies that HRQoL is lowered from 1 to 0,5 during ten years and then increases to 0,7 during ten years. B implies that HRQoL is lowered from 1 to 0,7 during the first ten years and then decreases to 0,5 during the next 10 years^4^. Now there are two new drugs on the market D_A_ and D_B_ able to target the conditions. Both improves the HRQoL with 0,2 units whenever it is used.

On the complete conditions view, the severity of A and B would be the same, and hence the acceptable cost-effectiveness of the two drugs should be the same. Is this reasonable? If we consider administering treatment at the breakout of A and B (and ignore any future discounting), based on the difference in severity we would have stronger reasons to treat condition A during the first ten years and then after ten years we have stronger reasons to treat B. Should this also be factored in to modify the acceptable cost-effectiveness level between D_A_ and D_B_? Since patients will be in different phases of A and B when the decision is made, and hence A-patients in their first ten-year phase and B-patients in their second ten-year phase will exist concurrently, it might seem reasonable to accept the same cost-effectiveness level for the two drugs and hence the same severity for the two conditions.

But should we take some other temporal perspective on A and B? Obviously, to take a forward-looking perspective on A and B at breakout of the two conditions, would result in the same severity as with the complete conditions approach. However, let us consider the assumption for why we should take severity into account: that patients are in an absolute state of worseness and therefore we have reason to improve their situation the worse off they are. Hence, the moral value of raising someone from 0,5 to 0,7 is higher than raising them from 0,7 to 0,9 and we should be willing to spend more relative resources doing so. Applying this to A and B, we could argue that we have reason to separate between different phases of A and B, where the first phase of A and the second phase of B have higher severity than the second phase of A and the first phase of B. This could then be handled by viewing A and B as consisting of four different conditions, with different severity and thus, different acceptable cost-effectiveness levels. However, this does not settle the question between the complete conditions and forward- looking view, it rather implies that we have reason to distinguish between different temporal stretches with different impact on HRQoL – in order to prioritize in line with the rationale for severity.

So, let us consider another situation, returning again to example 2. The new drug D_Y_ will improve the HRQoL from 0,5 to 0,7 as long as it is administered. With a complete conditions view, we have reasons to continue to administer D_Y_ through the full 35 years the condition last and at the same cost-effectiveness level. If instead, we consider a forward-looking perspective, the severity of Y will be reduced as the years goes by (since less of the total lowering of HRQoL will be in the future) and hence we should be willing to pay less and less for D_Y_. At some point during the 35 years, if the drug is priced close to the threshold, this will imply that the drug will be too costly to reimburse, and hence patients will not receive it. This despite being in the same situation in terms of HRQoL as before (if not receiving the drug). This does not seem to take the rationale for severity (the absolute worse-off-ness) seriously enough. Hence, we conclude that the forward-looking conditions perspective is not reasonable. Such a conclusion will also have implications for the issue of discounting severity (4). In discarding the forward-looking view, we will also discard of discounting. Since we look at the full, affectable stretch of a condition from the ‘outside’ so to speak, no part of this is in the future to be discounted.

A tentative conclusion at this point is that we have reason to adopt a complete-conditions view as long as the condition behaves in a monotonous way in terms of impact on HRQoL. If there are differences in levels of HRQoL over time, we have reason to divide the condition into different temporal stretches.

What if we have a condition with a monotonous impact on HRQoL, but the treatment can only affect part of this condition? Consider the following situation:

Example 4: Compare condition Y from example 2 with condition Z, where condition Z will imply a lowering of HRQoL from 1 to 0,5 during 10 years. None of these conditions have previous access to any treatment. At the market comes D_Y’_ and D_Z_ – both of which will improve the HRQoL of patients suffering from Y and Z with 0,2 units of HRQoL as long as it is used. However, given the physiological working mechanism of Y and how that changes over time, D_Y’_ will only be effective for Y during the last 10 years (of the total 35 years stretch).

Following our tentative conclusions, we would allow a much higher cost-effectiveness threshold to reimburse D_Y’_ than D_Z_ and thereby also a higher price, due to the difference in severity (17,5 vs 5,0) from a complete conditions’ perspective. Once again, this does not seem to take the worse-off-ness of patients with Z serious enough, since during the ten years when the drugs are effective they are in the exact same situation in terms of reduced HRQoL. We could perhaps have some stronger reasons to provide treatment to patients suffering from Y, but with the complete conditions view, the difference would seem to become too large. To test this further, imagine that a version of condition Z, call it Z’, instead would imply a HRQoL level of 0,3 during ten years. A complete conditions view would yield 17,5 and 7,0 for Y and Z’ respectively, resulting in a higher threshold to treat patients with Y than with Z’ which is contradictory to the rationale for taking severity into account. If we have the intuition that we should give some weight to the previous stretch, we would still end up with the same implication if the stretch is large enough. We draw the tentative conclusion, that even if intuitively reasonable when looking at individual lives, when applying severity as a modifier to cost-effectiveness thresholds, we should not take the temporal part of a condition we cannot affect with treatment into account. What do we mean by the affectable part of the condition? Is it only the temporal part of a condition that will actually be affected by a treatment or the part that can in principle be affected? Consider a modified version of example 4:

Example 5: Compare condition Y from example 2 with condition Z, where condition Z will imply a lowering of HRQoL from 1 to 0,5 during 10 years. None of these conditions have previous access to any treatment. At the market comes D_Y’’_ and D_Z_ – both of which will improve the HRQoL of patients suffering from Y and Z with 0,2 units of HRQoL as long as it is used. However, given the physiological working mechanism of Y and how that changes over time, D_Y’’_ will only be effective for Y during the first 10 years (of the total 35 year stretch).

Allowing the rest of the 35 years stretch of Y to be taken into account would imply that we have reason to accept a higher cost-effectiveness threshold for D_Y’’_ than for D_Z_. If we had to choose between providing relief for patients with Y or Z, it seems obvious we do have stronger reasons to provide relief for patient with Y, since they are facing a longer stretch of HRQoL-lowering. If we could choose between ending up in condition Y or Z, we would have rational reasons to choose Z, due to the lower severity. In effect, with affectable temporal stretch we do mean the temporal stretch we in principle can affect when treatment is considered, and not only the actual stretch we would affect.

With our approach to severity, the severity of Y and Z will be 17,5 and 5 respectively. Hence, we would be willing to accept a much higher cost-effectiveness threshold for Y than for Z. Let us now assume that we find a milder version of Y, called Y’, only implying a lowering to 0,7 during these 35 years resulting in a severity of 10,5. Consider again the worse version of Z, called Z’, with a lowering to 0,3 during 10 years, resulting in a severity of 7. With our approach to severity, the fairly mild condition would outweigh the very large impact on HRQoL, due to the longer temporal stretch, and of course this becomes even more pronounced when looking at a population that spends 70 years in a 0,1 lowering of HRQoL (which is basically where the normal population are in terms of HRQoL levels), which would equal 10 years of a very large impact. This is a more problematic implication of our approach.

One way to handle this is to introduce a threshold approach. Gustavsson and Juth have introduced this generally in priority setting [22], and it could be argued that in order to avoid some of the implications of the former section this could be a promising way forward. A double threshold approach to severity could then imply that we are not allowed to make trade-offs between different levels of HRQoL, hence even a very long stretch of limited HRQoL lowering could not trump (in terms of severity) the shorter stretch of a very high lowering (see Ehlert forthcoming). However, this would only handle some of the above problems, and the implication that we should be willing to spend more to achieve a positive health-effect on Y than on Z remains.

A tentative conclusion at this point is that we have reason to adopt a complete conditions perspective taking into account whole affectable stretches of time, to the extent they behave monotonous. If not, we might have reason to divide a temporal extension of a condition into different stretches, with different severity. In order to avoid unacceptable tradeoffs between degree of HRQoL impact and time, we might have reason to explore a double threshold approach to severity assessment.

Impact on Life-length.

Up until now, we have only looked at impact on HRQoL, and ignored impact on life-length. What if, we also consider impact on the length of life? Now, in line with many others, we will assume that a shorter life is generally worse than a longer life (as long as life is worth living, i.e. adds positive HRQoL to a life) (see for example [23]). Now consider example 6:

Example 6: Condition D implies instant, and premature death and will result in patients suffering from D losing 12 years of full HRQoL. Condition E implies instant, and premature death and will result in patients suffering from E losing 20 years of full HRQoL.

If we develop treatments for these two conditions, that will increase the life-length, we should be willing to accept a higher cost-effectiveness threshold for treatments targeting condition E than for D. Now, impact on life-length will, for obvious reasons, always lie in the future from the point in time when we consider treatment. Does it matter when in the future this will occur? Consider example 7 (adding information to example 6).

Example 7: Condition D affect people being 75 and hence the time between getting D and dying is short (if nothing is done). Condition E is congenital but will not affect the persons suffering from E until they are 65.

If we have a gene therapy D_E_ that will reduce the impact of E with ten years that has to be administered at birth, and a therapy D_D_ that will reduce the impact of D with ten years if administered at the outbreak of D, should this impact on the acceptable cost-effectiveness levels of the two therapies? Our complete affectable time-stretch conditions approach to severity imply that we should assess the severity of the part of the condition we can affect. In this case, we can affect the future loss of life-length, and our approach does not need to take into consideration when treatment has to be administered. That is, it is the part of a condition we can affect, not when we act in order to affect it that is interesting. But does this not raise a problem, not encountered when only looking at HRQoL-impact? We said that treatment for D and E will provide another 10 years of life. If so, is not the affected time-stretch the 10 years of lost life and hence the severity will be identical to the treatment effect? Let us expand a little on this.

With HRQoL, we assumed we had a reference level of 1 when there are no HRQoL issues or problems. The severity is the deviation from this level during the period in time we can affect by treatment. HRQoL and time here obviously move on different axes, so to say. When it comes to life-length, we also need a reference level to claim that a patient or patient population is worse off or has a condition with a certain severity in line with our non-person-relative conceptualization of severity. There has been some discussion about what this reference level should be during the last few years (see for example Ottersen [8]). Should it be, as suggested by the first Norwegian white paper on severity and absolute shortfall, 80 good life years [2]? Or, as the modified Norwegian suggestion, life-expectancy at the average age of when treatment should be administered for the patient population [17]? Both of these suggestions suffer from some problems. The first, that patients dying after having received 80 good life years (perhaps in their 90’s) do not have any severity at all, even if they could have lived on for yet some (good) years. The second suffers from the problem that the reference level will change depending on the age of the patient population. To use Norwegian life-expectancy tables, at birth the average life-expectancy is 82,42 years, at 82 the life-expectancy is 8,08 years^5^. Even if this discrepancy might be marginal, one way to handle this is to assume that the reference level is simply the (current) maximum life-expectancy of humans, which seems to be about 120 years [8]. With life-prolonging treatment, the affectable time-stretch is simply the stretch between patient’s death (without treatment) and the chosen reference level. What might make us draw the (wrong) conclusion that this is different from how we handle HRQoL, is that the aspect we can affect (life-length), move on the same axis as the time-stretch.

Some jurisdictions, e.g. Sweden [17], do apply a time to death criterion for severity. How should that be viewed from our affectable time-stretch approach to severity? Consider once again condition D. Assume that when someone gets D (at T_0_), the time to death (if nothing is done) will be 12 months. If we allow time to death to affect the overall severity (of losing 10 years), severity will increase for every month we wait to administer treatment to patient suffering from D. Hence, we would be willing to spend relatively more on the patient group for using D_D_ at T_3 months_, T_6 months_, T_9 months_ etc. To emphasize, regardless of the time to death, the patient will lose as many years in relation to the reference level, i.e. 10 years. Setting aside the problem of how to weigh time to death in relation to lost life-years, and ignoring the counter-intuitive implication that we should pay more for treatment the longer we wait to administer it – we will also have the following and perhaps more problematic counter-intuitive implication.

Example 8: Consider condition D where at T_12 months_ in relation to the time of administration of treatment, patients will lose 10 years. Compare this with condition F where at T_36 months_ patients will lose N years. If time to death is allowed to impact on severity, depending on the weight of time to death, condition F will have lower severity than condition D, despite *N* > 10 years.

In other words, a condition implying more years lost, will have lower severity than a condition with fewer years lost, if the latter condition has a shorter time to death. That seems simply wrong.

Should we discount future impact on life-length (in line with what NICE seems to do), even if we concluded that we should not do so for impact on HRQoL (4)? Let us say we apply a 3% yearly discount rate for future losses to the conditions D and E. For condition D this would imply that the total loss will be ≈ 8,79 years (instead of 10 years), whilst the loss of life for E will be almost eradicated (≈ 2,24 instead of 20 years). This does not seem reasonable from a complete-life’s perspective and would imply that we do value the life-expectancy of the 75-year-old group suffering from D as higher than for the newly born with E – despite the fact that they will die at a younger age. This does not seem reasonable.

It has been argued that this approach to life-length is age discriminatory. In this article we will not go into that issue, but refer to others who have, to our mind, convincingly shown, that it is not [24, 25]. Another problem is how to handle heterogenous populations, something we will ignore in this article (see footnote^6^).

In effect, we conclude that the complete affectable time-stretch conditions approach works also for impact on life-length. To sum up, in this section, we have argued that a complete affectable time-stretch conditions approach focusing on the period where treatments affect HRQoL and life-length is most promising and that time to death criterion and discounting future impact should be avoided.

## Shape of Conditions

As Davies and other notes, those arguing the need for having a time-relative view on distributive justice, never argues that it should be the only view, but rather be part of a pluralistic view on distribution [11]. We will now look at a few different shapes of conditions to see if that should be factored into our approach to severity assessment:

- Whether the impact is constant or intermittent.
- Whether the impact is increasing or decreasing.
- Whether there is a small difference in impact between conditions.
- Whether the impact is short but extreme.

### Every Now and Then

One issue to consider is if a condition constantly affecting a patient population or intermittently affecting a population impacts on its severity. Consider example 9:

Example 9: Condition G will imply a constant lowering from 1 to 0,5 during 1 year. Condition H will imply an intermittent lowering from 1 to 0,5, every other month during 2 years. In total, the time during which the populations are affected is the same.

In this case it is difficult to see why we should make a difference between conditions G and H in terms of severity: the fact that the effect on H is intermittent should not impact on our assessment per se. This is also the implication of our affectable time-stretch approach to severity, since the stretches when H is not affecting patients, cannot be affected by treatment and then should not be taken into account.

However, there are aspects of intermittence that might affect our assessment, but these are effects of intermittence rather than intermittence mattering in itself. For instance, intermittent impact of a chronic illness, may make the total effect less than with a continuous impact during the rest of life (unlike case 9, where impact is defined as the same for both persons). On the other hand, with continuous effect, the patient might adapt to the lowering in a way that might reduce the subjective impact, hence resulting in a somewhat higher HRQoL [26]. Intermittent effect might make it more difficult to adapt. Again, that would be a result of the effect of intermittence rather than this factor making a difference in itself. Assessing what effects intermittence have on HRQoL is an empirical matter that must be determined on a condition-by-condition basis.

### Ups and Downs

In example 2 we introduced condition Y implying a lowering of HRQoL from 1 to 0,5 during 35 years, which implied a total lowering of HRQoL of 17,5. Does it matter how this loss of 17,5 is distributed over life? Consider the following two conditions:

Example 9: Condition I implies an instant lowering of HRQoL to 0,8 at T_0_ and then an instant lowering of HRQoL after at T_17,5 years_ to 0,2 (a total loss of 17,5). Condition J implies a radical lowering to 0,2 at T_0_ and then an increase to 0,8 at T_17,5 years_ (a total loss of 17,5).

If we have two different treatments D_I_ and D_J_, that can be administered during the full 35 years, should we be willing to pay a higher cost per effect for one of these treatments? Psychological studies show that people seem to prefer a brightening future rather than a bleak one [27, 28]. Should that affect how we assess severity?

Accepting that shape makes a difference, at T_0_, I will be more severe than J and hence, we should be willing to pay more per effect for D_I_ than for D_J_ during the full 35 years. This implies that after 17,5 years, we should continue to pay more for D_I_, despite patients suffering from I is now in a situation with less impact on their HRQoL than patient suffering from J. This is contradictory to the rationale of taking severity into account.

Following our reasoning above, instead we have reason to distinguish between different time-stretches when the impact on HRQoL is different over time on a population. Hence, between T_0_ and T_17,5_ we should be willing to pay more to treat J, but during the rest of the time period, we should be willing to pay more to treat I and over time, this would imply that we would be willing to pay as much (on average) for the two treatments. If it turned out both I and J had a shorter period at the end (say 5 years) with a lowering to 0,2 or an increase to 0,8, respectively, we should obviously be willing to pay more for treating J on average since the total lowering is greater, in turn depending on J has a greater severity during a longer period. Once again, this is handled by our affectable time-stretch approach.

### Small Difference

Another issue to analyze under this part is how to account for small differences in lowering between different conditions. Consider example 10.

Example 10: We consider once again condition Y, with a 35 year lowering of HRQoL from 1 to 0,5. Compare this with condition K, with a 35 year + 5 day lowering of HRQoL from 1 to 0,5.

Is K worse than Y? Or, is the difference in 5 days a too small difference to actually make a difference.^7^ Now, obviously, in real life, the lowering of HRQoL from 1 to 0,5 might vary over time for both Y and K in a way that makes it difficult to claim that another 5 days for K is worse (since patients suffering from Y might have had stretches during the 35 years when they had a somewhat greater lowering or something of sorts). But let us try to ignore reality for now and focus on this case where we know that there is a difference of 5 days. First, we must assume that a lowering of HRQoL for 5 days is severe enough to be relevant to consider at all – implying that we would have reason to spend healthcare resources on trying to avoid this for a person (unless there were higher ranked competing conditions). If not, we can simply expand the 5 days until we find we have such reasons. In this context we assume we do have such reasons in example 10.

If this is true, this would also imply that if we have conditions Y and L, where L implied nothing but a 5 days lowering and there were no resource-related (or other) reasons against treating both Y and L we should treat both, even if Y would be considered to be a much more severe condition. This is a reason not to ignore the 5 days when we compare Y and K: why would the extra five days stop mattering when we are doing priority setting if it matters in general? Hence, it seems relevant to take into account also small differences between different stretches of times and assess K as being a slightly worse condition than Y. That we should take small differences into account when determining severity has also been argued elsewhere [30]. Once again, with a condition perspective, such small differences might not be of practical relevance – due to an acceptable temporal variation in one and the same condition. Still, relevant small differences between different conditions should be taken into account.

### Short but Extreme – or on using Severity Quantitatively

This leads us to another and related, but trickier, issue. Ottersen [8] identifies this as a problem for taking past health into account (i.e. the complete life’s approach). However, we contend that this is a problem for every approach that wants to quantify severity in relation to temporality. To illustrate, condition L in the former section implied a short stretch of HRQoL-lowering and would in itself imply a very low severity in relation to conditions like Y. In having to prioritize between such situations, it would seem our willingness to pay for treatment to affect L would be much lower than what we would be for Y. Is it reasonable to use severity in such a quantitative way? Consider an even more extreme case.

Example 11: Condition M implies an extreme pain during three hours, a situation interpreted as just over 0 (say 0,001) on our HRQoL-scale.

Since the duration of M is so short, the severity of M would be negligible compared to conditions like Y. At the same time, anyone having experienced this type of situations, would probably argue that we have strong reasons to also relieve such pain. One idea would be that there is a threshold effect, when a very high lowering of HRQoL should be given a ‘severity-premium’. However, in this case the severity-premium would be very high to compensate for the short duration and make the difference between the severity of Y and M more reasonable. To illustrate, without a severity premium, the 17,5 severity level of Y should be compared to a level of ≈ 0,00034 (3/24 × 365 × 0,999).

Let us zoom out and have a short look at how severity is used in the jurisdictions mentioned at the outset of the article. Norway, in using absolute shortfall, is using a quantified basis for severity, in terms of lost QALYs or lost good life years (a term they sometimes use). However, for practical (and ethical? ) reasons, this is translated into a three-level severity assessment (low, moderate and high) [2]. Anything equal to or higher than an absolute shortfall of 20 QALYs is assessed as the highest severity, and thereby implies the highest cost-effectiveness threshold (disregarding rare conditions and orphan drugs). This, in turn, implies that a large number of “severity differences” are not allowed to make a difference to the actual cost-effectiveness of treatment. We find similar approaches in the other jurisdictions we mentioned, Sweden, England/Wales, and the Netherlands. We know of no country using a quantified measurement of severity and using a monotonous function for the relation between severity and cost-effectiveness thresholds.

Obviously, it is not impossible to create such a function by identifying the highest possible severity achievable given certain assumptions. If we use our approach to HRQoL and use a 120 year reference level for life-expectancy, the highest possible severity would be to die at birth with a severity of 120. This in turn could be used to define the highest possible threshold for cost-effectiveness and then every severity level would be translated into a fraction of this level. One implication of this would be a very low willingness to pay for treatment to relieve people of condition M in example 11. We could then either bite the bullet of this or make an exception in order to be as consistent as possible for other conditions. There will be practical challenges with this approach and since there will be great uncertainties for how conditions will affect people, all severity assessment are likely to come with broad confidence intervals (even disregarding aspects like internal heterogeneity to conditions).

With the kind of broader severity categories used in actual healthcare jurisdictions, we avoid some of these problems. However, the result is that we do not take severity as serious as the underlying rationale would have us to do. To illustrate with the Norwegian approach, a difference in severity of 60 QALYs between two populations would be treated as on par in terms of the moral value of changing their respective situation for the better. If we do take severity seriously, it seems we have reason to find a practical application closer to the quantitative approach, despite some strange or problematic implications [17].

### Summing Up and Some Reflections on Implications for Practice

In this article we have explored how severity as a modifier for cost-effectiveness should be interpreted in terms of temporality. We have argued that we have reason to focus on conditions, rather than individuals. Furthermore, we have argued that when assessing the severity of a condition, we have reason to look at complete conditions from a time-neutral perspective, meaning that we take the full affectable stretch of the condition into account without modifying severity as patients move through the temporal stretch and without discounting the future. We find that this approach is also reasonable when considering impact on life-length. In considering different shapes of conditions, we conclude that even if some of these shapes could be relevant in individual lives, it is not relevant to take this into account when applying severity as a modifier for cost-effectiveness. The perspective we adopt on severity should, as far as possible, be applied with a quantified approach, in order for differences in severity to make a difference to decision-making.

Based on our experiences with decision-making with reimbursement of pharmaceuticals, we would also argue that the temporal approach to severity is applicable in practice. However, we do agree with Herlitz [12], when he claims that suggestions for a specific temporal perspective raise practical challenges with ethical aspects:

Is it possible to predict people’s future health status? How should this be done? This will plausibly depend on the circumstances. Certain illnesses have predictable trajectories, while others do not [12]. , p. 526.

To this could be added: how should we handle heterogenous conditions where there is great variation in impact on HRQoL and life-length, and where this cannot be solved by distinguishing between different more monotonous stretches of conditions? Should we allow priority exceptions to individuals with outlier severity in relation to the established severity for the condition? Should we allow exceptions to conditions, like the extreme but short impact on HRQoL discussed above, not able to fit into the more general scheme? Etc. Hence, there are ample unresolved issues on severity for further exploration.

## Footnotes

1 Hence, we ignore all issues related to the badness of death. We do assume that there is a conceptual link between being worse off and having a severe illness: the more severe the illness, the worse off is the individual and conversely [5].

2 Quality-Adjusted Life Year.

3 One temporal aspect we will bracket in this context, is whether different stretches of life, like childhood, have different value to a human life (see for example McMahan et al. 2009 and Kerstein and Bognar 2010 for a detailed discussion [20, 21]).

4 At this point we will ignore whether we have reason to consider the shape of this condition, see below.

5 Unpublished working material.

6 Focusing on conditions that might affect people of different ages, we have different choices. In order to arrive at a more accurate severity assessment, we might divide the conditions into different age categories to account for the fact that how large the life-length loss is, is dependent on in which age you get the condition. Or we might simply take the average life-length loss of the condition. This latter approach will ‘benefit’ patients getting the condition later in life (in relation to the average), and ‘disadvantage’ patients suffering the condition earlier in life (in relation to the average). If this spread of age is large, the beneficial or disadvantageous effect might be substantial. However, at this point we will not go further into this discussion.

7 This line of reasoning has been suggested by e.g. Frances Kamm [29].
